# The fascinating and secret wild life of the budding yeast *S. cerevisiae*

**DOI:** 10.7554/eLife.05835

**Published:** 2015-03-25

**Authors:** Gianni Liti

**Affiliations:** Institute for Research on Cancer and Ageing of Nice, CNRS UMR 7284, INSERM U1081, University of Nice Sophia Antipolis, Nice, France

**Keywords:** the natural history of model organisms, natural history, population genomics, *S. cerevisiae*

## Abstract

The budding yeast *Saccharomyces cerevisiae* has been used in laboratory experiments for over a century and has been instrumental in understanding virtually every aspect of molecular biology and genetics. However, it wasn't until a decade ago that the scientific community started to realise how little was known about this yeast's ecology and natural history, and how this information was vitally important for interpreting its biology. Recent large-scale population genomics studies coupled with intensive field surveys have revealed a previously unappreciated wild lifestyle of *S. cerevisiae* outside the restrictions of human environments and laboratories. The recent discovery that Chinese isolates harbour almost twice as much genetic variation as isolates from the rest of the world combined suggests that Asia is the likely origin of the modern budding yeast.

**DOI:** http://dx.doi.org/10.7554/eLife.05835.001

## Introduction

Humans have exploited the budding yeast, *Saccharomyces cerevisiae*, for over ten thousand years for brewing and baking. This close connection with human activity led Louis Pasteur to discover its essential role in alcoholic fermentation in 1857 (Pasteur, 1858). Brewing was also the key motivation for the start of yeast genetics. The initial breeding experiments by Ojvind Winge at the Carlsberg laboratory in the 1930s aimed to combine desirable brewing traits by crossing different strains (Barnett, 2007). Our ability to control and manipulate its life cycle has made the budding yeast the most powerful, single-cell eukaryotic system for biological research, and it was rapidly adopted around the world to investigate virtually every aspect of biology. Early on in *S. cerevisiae* research, the scientific community adopted a single reference isolate, the S288c strain, or strains derived from it. This reference strain was obtained by crossing several parental strains and has the peculiar trait that it can be maintained with a stable haploid background, making it easier to study the effects of mutations (Mortimer and Johnston, 1986). In 1996, S288c became the first eukaryotic organism to have its genome completely sequenced (Goffeau et al., 1996), and strain libraries were subsequently developed for it, such as libraries of deletion (Giaever et al., 2002) and overexpression mutants (Sopko et al., 2006), and strains with genes tagged by reporter genes (Huh et al., 2003). These libraries allow researchers to investigate the functional biology of every *S. cerevisiae* gene. The availability of such a powerful functional genomics toolkit has facilitated some remarkable discoveries, such as defining the yeast genetic and protein interaction networks (Botstein and Fink, 2011).

However, this reference strain provides very little information about the natural history of this species, and its combinations of alleles have never been exposed to selection in a natural setting. This strain is also an outlier in terms of its phenotypic properties (Warringer et al., 2011), and the presence of auxotrophic markers (i.e., mutations that render a yeast cell unable to synthesize an essential compound) in its genome has serious consequences for many traits (Mülleder et al., 2012). In the past decade, we have witnessed a renewed interest in understanding the fascinating secret life of the budding yeast. On-going studies of thousands of isolates, both wild and those associated with human activities, will reveal the impact humans have had on the evolution of this species and will help to keep *S. cerevisiae* at the forefront of systems genetics.

## Lifecycle outside of the lab

**Figure 1.**
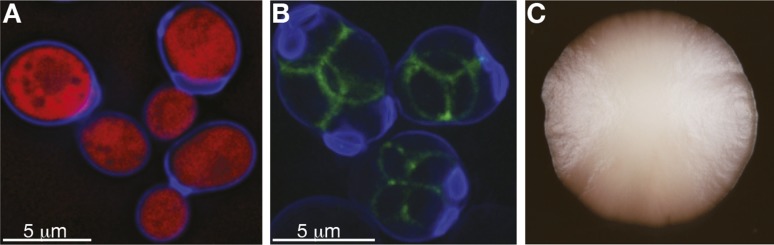
*S. cerevisiae* yeast: single cells and as a population. (**A**) Budding cells of the reference (S288c) strain of *S. cerevisiae*, expressing red fluorescent protein (RFP), which marks the centre of the cell. The cells are also stained with calcofluor-white, which stains the outer walls of the cells blue. (**B**) Sporulating cells of the North American oak *S. cerevisiae* isolate YPS606, stained with calcofluor-white, in which the protein *SPS2* is labelled with GFP (Gerke et al., 2006), which marks the ascospore wall in green. (**C**) A clonal colony with a population size of approximately 7 × 10^6^ cells derived from a single *S. cerevisiae* cell grown on a solid agar medium. Such colony structures have never been observed in the wild. Image credits: Benjamin Barré and Gianni Liti. **DOI:** http://dx.doi.org/10.7554/eLife.05835.003

The *S. cerevisiae* lifecycle, under precisely controlled laboratory conditions, is one of the best understood at the mechanistic level. Our ability to control its sexual cycle and to switch it between mitotic and meiotic reproduction is one of the great experimental strengths of this yeast system. Paradoxically, we know very little about its life cycle in natural settings (Box 1), and what little we do know is indirectly inferred from studying its life cycle in the lab, which precludes any firm conclusions (Boynton and Greig, 2014). In the wild, yeast cells are found in fluctuating environments and are often subjected to a shortage of food. For instance, one of this yeast's natural habitats, oak bark, subjects it to seasonal cycles of tree sap flow, in addition to changing climate conditions. *S. cerevisiae* cells therefore likely spend much of their time in a non-dividing state called quiescence (Gray et al., 2004). When conditions become favourable, *S. cerevisiae* is able to grow on a modest array of fermentable and non-fermentable carbon sources (mostly six-carbon sugars). The availability of nutrients is likely to result in a rapid, mitotic clonal expansion of diploid yeast cells (Figure 1A,C).

10.7554/eLife.05835.002

Box 1. **Outstanding questions about the natural history of *S. cerevisiae***

- How does the *S. cerevisiae* life cycle progress in the wild?

What is the importance of the different life-cycle phases and of sexual reproduction? New insights into these questions could be obtained by directly observing natural populations or by analysing bar-coded strains released in a controlled wild environment.

- Can we assemble an exhaustive catalogue of *S. cerevisiae* ecological niches?

Systematic surveying would reveal what this yeast's natural habitats are and the substrates on which it lives. This would help us to better understand the environmental selective pressures that shape it, and would allow gene function to be tested in ecologically relevant conditions.

- How does *S. cerevisiae* interact with other microbes in its natural habitats?

The structures of coexisting microbial communities and their population sizes are often problematic to resolve, due to the reliance of an enrichment step when isolating *S. cerevisiae* natural populations, which favours its growth over that of other microbes. Metagenomic approaches, involving the sequencing of natural samples, might reveal in a quantitative manner the interplay of *S. cerevisiae* with other microbial communities.

- What is the extent of *S. cerevisiae* genome variation beyond SNPs?

Most genomic surveys of *S. cerevisiae* have focused on genetic distance based on SNPs and have been limited to canonical diploid strains (or their derivatives). Future studies should also determine the prevalence of aneuploidies and of ploidy variation. However, the repetitive subtelomeric regions of *S. cerevisiae*, where structural variation can be enriched, remain technically challenging regions to sequence, analyse and assemble correctly. New long-read sequencing technologies could help to overcome this problem.

**DOI:** http://dx.doi.org/10.7554/eLife.05835.002

Sexual cycles can be triggered by environmental cues, such as nutrient depletion, and result in the production of four meiotic spores that have two distinct mating types (a and α), analogous to human eggs and sperms (Figure 1B). Meiotic spores tested in laboratory experiments are highly resistant to various stresses, such as high and low temperature and desiccation, and it is reasonable to assume that in a wild habitat they can persist for long periods of time until favourable, nutrient-rich conditions allow germination. Haploid spores eventually re-establish diploid lines, either by mating with their own mitotic daughter cells after switching mating type (haplo-selfing), by mating with another spore created by the same meiotic event (intra-tetrad mating) or, more rarely, by mating with an unrelated individual (outcrossing) (Knop, 2006). These different mating solutions should have very different effects on species evolution and population structure. However, due to the microscopic size of yeast, the relative frequency of different modes of reproduction in the wild can only be inferred retrospectively by genome analysis (Ruderfer et al., 2006; Tsai et al., 2008). Population genomic studies indicate that budding yeast mostly reproduces asexually and that outcrossing is rare. Experiments have shown that outbreeding can be promoted by another popular model organism, the fruit fly *D. melanogaster*, which is attracted to and consumes yeast cells living on fruit in the wild and thereby acts as a vector, aiding their geographical dispersal (Reuter et al., 2007; Buser et al., 2014; Christiaens et al., 2014). Furthermore, outcrossing is not restricted to mating within the species; introgressed genomic regions and interspecies hybrids with other closely related species are frequently observed (Liti et al., 2006; Novo et al., 2009). *S. cerevisiae* belong to a group of species named the *Saccharomyces sensu stricto* complex, which can generate viable hybrids when interbred (Liti and Louis, 2005; Replansky et al., 2008; Hittinger, 2013; Borneman and Pretorius, 2015). One fortunate example of this is the hybridization between *S. cerevisiae* and *S. eubayanus*, which led to the hybrid species *S. pastorianus,* used worldwide in the brewing industry to produce lager (Libkind et al., 2011).

## A growing range of ecological niches

**Figure 2.**
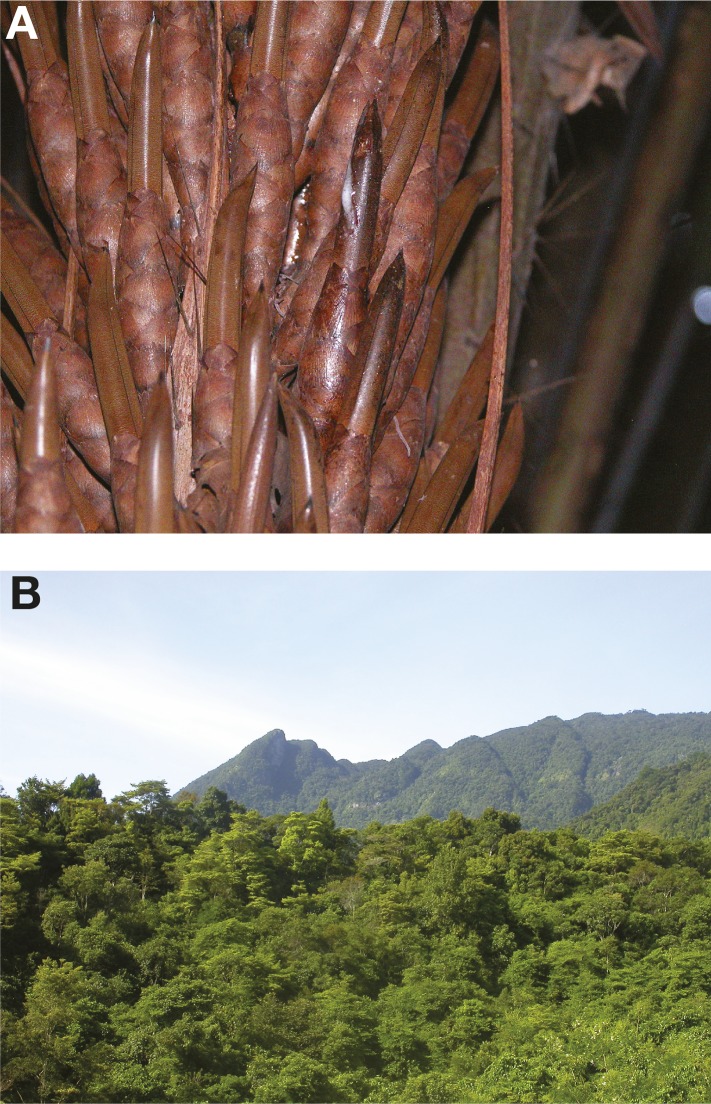
Emerging wild ecological niches of *S. cerevisiae*. (**A**) A close-up of a fermenting bertam palm bud; the Malaysian *S. cerevisiae* lineage has been isolated from these palms (Wiens et al., 2008). Malaysian *S. cerevisiae* strains are reproductively isolated due to a complex series of rearrangements. (**B**) A primeval rainforest in the tropical highland of Hainan where the highly diverged Chinese lineages (CHN-I, CHN-III and CHN-V) have been isolated (Wang et al., 2012). Image credits: (**A**) Marc-André Lachance; (**B**) Feng-Yan Bai. **DOI:** http://dx.doi.org/10.7554/eLife.05835.004

The abundance of *S. cerevisiae* associated with fermented beverages initially gave rise to the notion of a ‘man-made organism’, restricted to human settings (Vaughan-Martini and Martini, 1995). The chemical composition of the fermentation environment can vary between different types of beverage, and also throughout the fermentation process. This substrate variability has selected for different yeast breeds that are optimised to ferment specific products, such as wine and sake (Fay and Benavides, 2005). Additionally, several genome signatures of human-driven adaptation have been reported (Sicard and Legras, 2011). However, all fermented beverages adhere to the basic rules of alcoholic fermentation, with different types of sugars being rapidly transformed into ethanol. This metabolic trait has independently evolved in yeasts multiple times, perhaps as an adaptive strategy either because producing high concentrations of ethanol can help to outcompete other microorganisms (Rozpędowska et al., 2011; Thompson et al., 2013) or because it can support faster growth than aerobic fermentation (Pfeiffer et al., 2001). The alcoholic fermentation of sugar-rich substrates by *S. cerevisiae* and by other yeast species is not exclusive to human beverages. Ethanol content almost equivalent to that of lager beer has been detected in the spontaneously fermenting nectar of the bertam palm in the Malaysian rainforests, although it has not been determined if *S. cerevisiae* is the dominant fermenter in this context (Figure 2A). This alcoholic nectar is heavily consumed by the pen-tailed treeshrew (Wiens et al., 2008).

Apart from the human-associated environments of breweries and bakeries, another set of *S. cerevisiae* strains has an even more intimate association with humans. These include pathogenic strains isolated from immunocompromised patients and strains associated with *Candida* infections (Muller et al., 2011). Clinically relevant strains of *S. cerevisiae* can grow at high temperatures (above 40°C), perhaps as a result of adaptation to human body niches. A surprising case of non-pathogenic human colonization was recently reported from an Amerindian community living in a remote area of French Guiana. In this cohort, *S. cerevisiae* is frequently isolated from stools, in contrast to industrialised countries where *Candida albicans* is predominant (Angebault et al., 2013). This finding adds to a growing list of *S. cerevisiae* detected in the human microbiota of both healthy and sick individuals (Rizzetto et al., 2014). Colonizers are likely derived from food and beverages and appear to be able to persist in the human gut environment. Such resistance to the human gut is also observed in a variant of *S. cerevisiae*, known as *S. boulardii*, which is marketed as a probiotic.

In addition to human-related environments, *S. cerevisiae* has also been isolated from wild habitats. This is not a trivial task given the small population size present in nutrient-poor substrates. The sampling approach used relies on an enrichment medium that favours the growth of *S. cerevisiae* over other microbes that coexist in the sample. Oak trees (*Quercus spp.*, *Fagaceae* family) represent a natural niche where *S. cerevisiae*, together with closely related species of the *sensu stricto* complex, has frequently been sampled across different continents (Sniegowski et al., 2002; Sampaio and Gonçalves, 2008). Additional niches include other trees belonging to the *Fagaceae* family (such as beech and chestnut), other plants with completely different geographic ranges (e.g., cactuses), and associated soil and insects (Liti et al., 2009; Stefanini et al., 2012; Naumov et al., 2013). Perhaps the most successful field survey has been carried out across China (Wang et al., 2012). This study revealed for the first time that *S. cerevisiae* has a rather ubiquitous distribution, one that is not limited to human-associated environments but extends into other habitats, such as primary forests that are remote from human activity (Figure 2B). These new studies are challenging our concept of the natural niches of this yeast, by showing that its history goes far beyond its association with humans. Additional systematic field surveys will help to further refine our knowledge of its complex ecology (Box 1).

## Population genomics of wild and domesticated lineages

**Figure 3.**
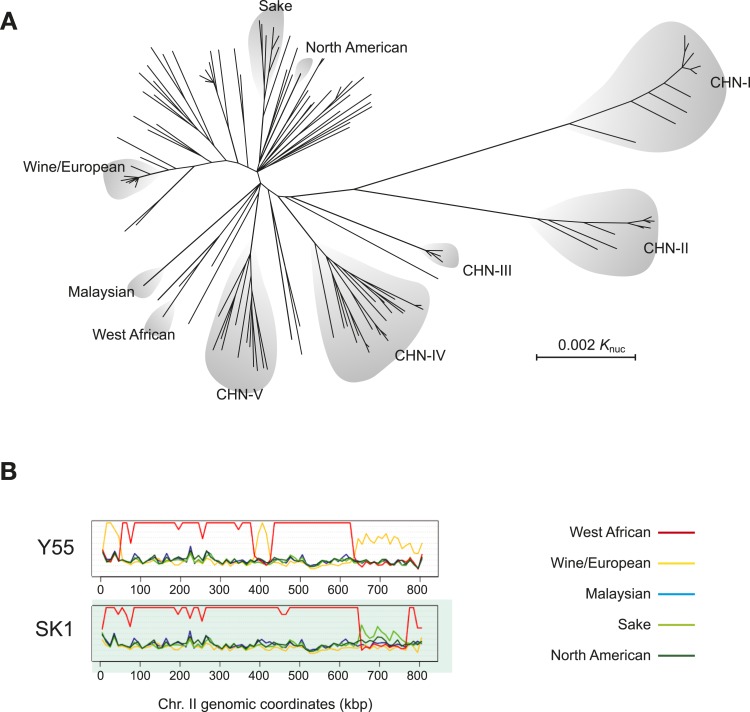
*S. cerevisiae* genome relationships and population structure. (**A**) A phylogenetic tree of *S. cerevisiae* isolates, adapted from (Wang et al., 2012). The main worldwide and Chinese lineages (denoted, CHN I-V) are highlighted. Lineages CHN-I, CHN-III, CHN-V were mostly isolated from *Fagaceae* trees in the rainforest of the tropical Hainan Island in the South China Sea. Lineages CHN-II and CHN-IV were isolated from temperate areas in north China (Shaanxi province and Beijing province, respectively). (**B**) Sequence similarity plots of the Y55 and SK1 laboratory strains showing the relationships to the five, worldwide genetically distinct lineages (listed on the right) along chromosome II. Similarity is defined as N/(D+1), where N is the number of positions in a chromosomal window and D is the number of those positions where the nucleotides differ. The genomes of both Y55 and SK1 are mostly derived from the West African lineage (red line). However, large blocks of the chromosome show drops in similarity to the West African lineage and higher similarity to other lineages. Y55 has three segments (0–50 kb, around 400 kb, from 650 kb to the right telomere) with high similarity to the Wine/European genome (yellow line). Likewise, SK1 has a large segment (650–750 kb) with higher similarity to the Sake lineage (light green line). This type of analysis has revealed the mosaic genome structure of many *S. cerevisiae* isolates (Liti et al., 2009). Image credit: Anders Bergström and Gianni Liti. **DOI:** http://dx.doi.org/10.7554/eLife.05835.005

Population genomics has provided a powerful means by which to illuminate the evolutionary history of budding yeast. In initial genome sequencing studies, half of the *S. cerevisiae* strains sequenced fell into a number of distinct lineages (Figure 3A). Genetic variants within these lineages are mostly unique to a subpopulation and absent in others and evenly distributed across the genome (Liti et al., 2009). Variation in phenotype tends to follow population structure (Warringer et al., 2011). Some of these lineages are characteristic of distinct fermentation processes and might represent examples of domesticated breeds (Fay and Benavides, 2005; Schacherer et al., 2009). These strains do not strictly follow geographic boundaries, for example, wine strains from Europe, Australia, Chile and New Zealand share recent ancestry and reflect human migration history (Legras et al., 2007; Liti et al., 2009; Goddard et al., 2010; Dunn et al., 2012).

Other lineages are not associated with human activity and appear to be characteristic of the geographic area. These include oak strains from woodlands in North America and bertam palm strains from the Malaysian rainforest. The Malaysian lineage has the peculiarity of being reproductively isolated from all other lineages due to a complex series of chromosomal rearrangements (Cubillos et al., 2011; Marie-Nelly et al., 2014).

Although full genome information is not yet available for Chinese isolates of *S. cerevisiae*, they appear to exhibit strong population structure, with essentially double the combined amount of genetic variation identified in *S. cerevisiae* isolates sampled from the rest of the world (Wang et al., 2012). Chinese isolates from primeval forests fall into ancient and remarkably diverged lineages (Figure 3A). These results suggest that China harbours a reservoir of *S. cerevisiae* natural genetic variation, which perhaps gives an indication as to where the species originated. This enrichment of genetic diversity is not limited to *S. cerevisiae* intra-species variation as Far East Asia is also the only region where all the *Saccharomyces sensu stricto* species have been isolated.

In addition to these distinct lineages, many *S. cerevisiae* strains have been found, after sequencing, to have mosaic recombinant genomes, which reflect a mixed ancestry likely originating from outcrosses between genetically distinct lineages (Figure 3B). Mosaic strains have probably arisen as result of human activities (Liti et al., 2009). Clinical isolates and those used in bakeries and in the laboratory tend to be mosaics, and most of their genomes can be traced to already characterized genetically distinct lineages. Future population genomics studies should further define the genomic features of *S. cerevisiae* and allow the hybridisation events that generated these mosaic strains to be reconstructed.

## Future perspectives

Recent insights into the natural history of *S. cerevisiae* have shown that the reference lab strains represent only a subset of the many aspects of natural yeast life. Population genomics offer a powerful approach, termed ‘reverse ecology’ (Li et al., 2008), to investigate the natural history of the species. Population-level sequencing applied to thousands of strains will further illuminate the natural history of *S. cerevisiae*, and the genomes of highly diverged lineages that predate domestication will reveal the impact of human activity on the species. Analysing large numbers of strains also offers the opportunity to directly test associations between genotype and phenotype. Some lineages have specific genomic signatures that underlie phenotypes (Skelly et al., 2013) but whether this is driven by adaptation or by genetic drift remains an open question (Warringer et al., 2011). Genotype-phenotype associations are not restricted to single nucleotide polymorphisms (SNPs) but can extend to genome content, copy number, ploidy and structural variation (Box 1). Recent reports have shown that accessory genes vary between lineages and constitute a large fraction of genome variation between individuals (Bergström et al., 2014). Certain forms of non-genetic variation, such as prions, might also be important determinants of phenotypic variation (Jarosz et al., 2014). The legacy from classical genetics also offers possibilities to develop new tools and methodologies. Artificial populations of recombinant strains are now available, including a multi-parent population derived from highly diverged lineages (Cubillos et al., 2013). These approaches promise to bring yeast to the forefront of complex trait analysis together with other model systems where multi-parent populations are already established (Kover et al., 2009; Aylor et al., 2011; King et al., 2012).

In summary, a better knowledge of the natural history of this species is essential for interpreting its biology. Identifying how the *S. cerevisiae* life cycle progresses in the wild will allow us to better understand the genetic and environmental factors that are relevant to its fitness (Box 1). Yeast fitness has been largely approximated to mitotic growth, which disregards mortality during periods that do not support reproduction. However, it is likely that yeasts in nature spend most of their chronological life in a non-dividing state. The ability to survive in that state is therefore probably exposed to strong selection. Thus, measuring the impact of natural genetic variation on longevity related traits might be crucial for understanding the selective pressures that yeasts are exposed to during their life cycle. Consideration must also be given to other aspects of the life cycle, such as sporulation, spore viability and germination time, each of which are also complex traits and strongly affected by the environment. Exploiting the natural variation of *S. cerevisiae* and its close relatives using genomics methodologies is enabling a new era of functional and evolutionary genetics studies of this classic model organism (Nieduszynski and Liti, 2011; Fay, 2013; Lehner, 2013).
